# Impact of nucleic acid amplification test on pulmonary tuberculosis notifications and treatments in Taiwan: a 7-year single-center cohort study

**DOI:** 10.1186/s12879-019-4358-8

**Published:** 2019-08-16

**Authors:** Chih-Wei Wu, Yao-Kuang Wu, Chou-Chin Lan, Mei-Chen Yang, Ting-Qian Dong, I-Shiang Tzeng, Shu-Shien Hsiao

**Affiliations:** 1 Division of Pulmonary Medicine, Taipei Tzu Chi Hospital, Buddhist Tzu Chi Medical Foundation, New Taipei City, Taiwan; 2 Division of Infection Control, Taipei Tzu Chi Hospital, Buddhist Tzu Chi Medical Foundation, New Taipei City, Taiwan; 3 Division of Research, Taipei Tzu Chi Hospital, Buddhist Tzu Chi Medical Foundation, New Taipei City, Taiwan; 4 Division of Nursing, Taipei Tzu Chi Hospital, Buddhist Tzu Chi Medical Foundation, New Taipei City, Taiwan

**Keywords:** Nucleic acid amplification test, Treatment initiation, Pulmonary tuberculosis, Notification, Prescription error

## Abstract

**Background:**

Nucleic acid amplification tests (NAAT) have been used as a diagnostic tool for pulmonary tuberculosis (PTB) in Taiwan for many years. In accordance with Taiwanese legislation, health care personnel are required to notify the Centers for Disease Control and Prevention (CDC) in case of suspected PTB. This study aimed to investigate the impact of NAAT(Gen-Probe) on the notification system for PTB and anti-tuberculosis treatments in Taiwan.

**Methods:**

A retrospective study on the impact of NAAT (Enhanced Amplified *Mycobacterium tuberculosis* Direct Test [E-MTD], Gen-Probe, San Diego, CA, USA) [NAAT(Gen-Probe)] was carried out at Taipei Tzu Chi Hospital, Buddhist Tzu Chi Medical Foundation from March 2011 to December 2017. During the study period, microscopic acid-fast-bacilli smears and mycobacterial cultures were available for PTB diagnosis. NAAT(Gen-Probe) was first introduced at the hospital in January 2014 for use as a diagnostic method for PTB. Positive sputum culture was considered as the gold standard for PTB diagnosis. We excluded clinically-diagnosed PTB cases.

**Results:**

When NAAT(Gen-Probe) was applied, the rate of error notification to CDC decreased from 64.3 to 7.0% (*P* < 0.001), and unnecessary anti-TB treatments administered to suspected cases decreased from 14.9 to 6.5% (*P* = 0.005). In the non-PTB group, the mean duration of unnecessary anti-TB treatments changed from 38.9 ± 38.3 days to 37.0 ± 37.9 days (*P* = 0.874). In the PTB group, the mean time from notifying CDC to initiating treatment decreased from 3.05 ± 6.95 days to 1.48 ± 1.99 days (*P* = 0.004). The sensitivity, specificity, positive predictive value, and negative predictive value of NAAT(Gen-Probe) were 99.0, 92.3, 99.0, and 92.3%, respectively.

**Conclusions:**

Use of NAAT(Gen-Probe) led to decrease in the rate of error notification of suspected PTB cases to the CDC, avoidance of unnecessary use of anti-TB treatments, and accelerated initiation of appropriate treatments.

## Background

Tuberculosis (TB) persists as an important cause of morbidity and mortality in Taiwan, despite vigorous government effort. In 2017, a total of 9759 cases of TB were reported in Taiwan, of which, 511 patients died [1]. Diagnosis of TB typically relies on the detection of *Mycobacterium tuberculosis* (MTB) by mycobacterial culture. Acid-fast-bacilli (AFB) smear microscopy is inexpensive and rapid, but cannot distinguish nontuberculous mycobacteria (NTM) from MTB. Culture is more sensitive, but the results are obtained after several weeks [2]. Nucleic acid amplification tests (NAATs) represent a novel advancement in the diagnosis of TB and have been available in Taiwan for over two decades. NAATs constitute a rapid and sensitive method for diagnosing TB and are also useful in excluding infections caused by NTM [3].

The Centers for Disease Control and Prevention (CDC) in the US recommend routine use of NAATs as standard practice [4]. The Food and Drug Administration has approved several NAATs including Amplified *Mycobacterium tuberculosis* Direct Test [NAAT(Gen-Probe)], Amplicor *Mycobacterium tuberculosis* test [NAAT(Amplicor)], and Xpert MTB/RIF assay [NAAT(Xpert)], etc. for MTB diagnosis in the US [5]. The Enhanced Amplified *Mycobacterium tuberculosis* Direct Test (E-MTD), is based on transcription-mediated amplification of the 16S rRNA gene [6, 7] and approved for use in detecting MTB complex bacteria in AFB smear-positive and negative respiratory specimens from suspected cases of pulmonary TB (PTB). The ultimate goals of any TB control program are to reduce morbidity and mortality among patients and prevent disease transmission. Hence, early detection and treatment of cases has been a pillar for effective TB strategy [8].

The diagnosis of PTB in Taiwan includes the following: (1) chest X-ray findings that are compatible with PTB, (2) positive AFB smear, (3) positive MTB culture, (4) tissue histology revealing granulomatous inflammation, and (5) a positive NAAT. Physicians notify Taiwan CDC about cases of PTB according to any one of the above criteria and their own clinical judgments.

Many studies have highlighted the importance of NAATs. Use of these tests enable the reduction of unnecessary airborne isolation [9, 10], shorter duration of unnecessary anti-TB treatment [9, 10], and shorter delays to treatment initiation [11, 12]. In this study, the impact of NAAT using E-MTD (Gen-Probe, San Diego, CA, USA) [NAAT(Gen-Probe)] at a single center over a period of 7 years was retrospectively assessed; the following aspects were investigated: whether NAAT(Gen-Probe) (i) reduced error notifications of suspected cases of TB to Taiwan CDC; (ii) avoided administration of unnecessary anti-TB treatments; (iii) reduced the duration of unnecessary anti-TB treatments; and (iv) hastened treatment initiation. In addition, the sensitivity, specificity, positive predictive value (PPV), and negative predictive value (NPV) of NAAT(Gen-Probe) were evaluated.

## Methods

A retrospective study on the impact of NAAT(Gen-Probe) was conducted at Taipei Tzu Chi Hospital, Buddhist Tzu Chi Medical Foundation from March 2011 to December 2017. The aim of the study was to evaluate the impact of NAAT on patients with PTB, and therefore, patients with extra-pulmonary tuberculosis were excluded. The study focuses on bacteriologically-confirmed PTB. Thus, we excluded the clinically-diagnosed PTB such as cases with granulomatous lung disease or tree-in-bud pulmonary infiltrate. Because we excluded clinically-diagnosed PTB, both the correct or error notifications and necessity of treatment depended on bacteriologic culture results. Positive sputum culture was considered as the gold standard for PTB diagnosis. Sputum was considered as the only clinical pulmonary specimen, and bronchoalveolar lavage and lung biopsy specimens were excluded. Molecular diagnosis of PTB was carried out using NAAT(Gen-Probe). NAAT(Gen-Probe) was first introduced in our hospital in January 2014. From March 2011 to December 2013, AFB smears or MTB cultures were used by the physicians for microbiological diagnosis of PTB; in contrast, from January 2014 to December 2017, NAAT(Gen-Probe), AFB smears, or MTB cultures were used. NAAT(Gen-Probe), AFB smears, and MTB cultures were performed in the laboratory daily (excluding weekends).

The Mycobacteria Growth Indicator Tube (MGIT) system was introduced at the study hospital in March 2011 and has since been used routinely for mycobacterial culture. If MGIT showed growth of Mycobacterium species, the immunochromatographic diagnostic test (ICT diagnostics, Sydney, Australia) was performed to differentiate between MTB and NTM.

The study population included a total of 667 patients. Using the import date of NAAT(Gen-Probe), the study population was divided into two groups: Group A and Group B. PTB testing comprised two aspects: first, Group A and Group B were compared to assess the impact of NAAT(Gen-Probe); second, the diagnostic performance of NAAT(Gen-Probe) was described for cases in Group B, including sensitivity, specificity, PPV, and NPV. The diagnostic performance of NAAT was calculated by considering culture as reference standard.

The division of infection control at our hospital collected the data of suspected PTB cases and notified the Taiwan CDC. The data included the patients’ demographic data, laboratory data, treatment initiation dates, and treatment termination dates. The division of infection control performed follow-up of the notified cases at the outpatient and inpatient departments.

### Statistics analysis

Statistical Package for the Social Sciences (SPSS) version 24 software was used. Chi-squared or Fisher’s exact tests (two-tailed) were performed to assess differences in proportions. Wilcoxon rank-sum test was used to compare differences in mean time frames. Continuous data are expressed as the mean ± standard deviation, while categorical data are expressed as frequencies and percentages. A *P*-value of < 0.05 was considered to indicate statistical significance for comparisons. Open-access software, R version 3.4.3 was used to calculate the sensitivity, specificity, PPV, and NPV of NAAT(Gen-Probe). Binary data were also analyzed.

## Results

**Fig. 1:**
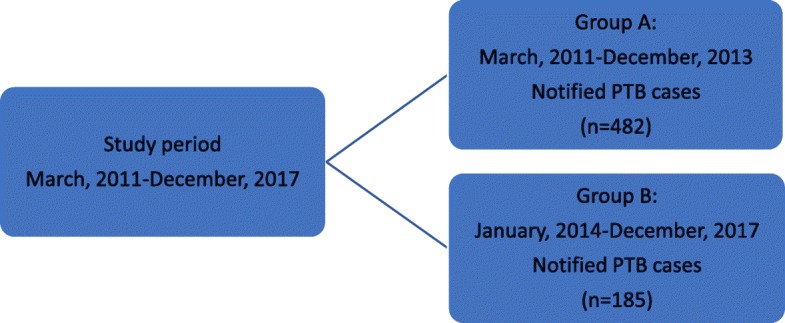
Flow chart of the study population before and after performing nucleic acid amplification tests (NAAT), Taiwan. * Notified PTB case: The case with suspected PTB reported to Taiwan CDC. * Confirmed PTB case: The case with positive MTB culture of the sputum

Flow chart of the study population before and after initiation of NAAT(Gen-Probe) is shown in Fig. 1. Group A comprised 482 patients from March 2011 to December 2013, prior to the introduction of NAAT(Gen-Probe), for whom notifications of suspected PTB were sent to Taiwan CDC. Group B comprised 185 patients from January 2014 to December 2017, following the introduction of NAAT(Gen-Probe), for whom notifications of suspected PTB were sent to Taiwan CDC.

The basic profiles of patients in Group A and Group B are listed in Table 1. The mean age of patients in Group A was 65.7 years and that in Group B was 62.4 years; the ages of the patients were not significantly different between the two groups (*P* = 0.051). Moreover, the sex distribution was not significantly different between the two groups (*P* = 0.647).

**Table 1:** Basic characteristics of the study groups

Basic profile	Group A	Group B	*P* value
Notified cases	482 cases	185 cases	
Mean age (y)	65.7 ± 18.0	62.4 ± 20.1	0.051^b^
Age groups			
0–18 (y)	8 (1.7%)	2 (1.1%)	0.125^a^
19–64 (y)	188 (39.0%)	88 (47.6%)	
≥65 (y)	286 (59.3%)	95 (51.3%)	
Male/female	299 cases/183 cases	119 cases/66 cases	0.647^a^

^a^Using Chi-squared or Fisher’s exact tests (two-tailed)

^b^Using Wilcoxon rank-sum test

The reasons for notification are listed in Table 2. In Group A, there were 431 cases notified to Taiwan CDC according to positive AFB smear and finally 121 cases had positive MTB culture. In Group B, cases notified by positive AFB smear decreased to 13 cases. The 13 cases all had negative NAAT(Gen-Probe) tests and finally only 1 case had positive MTB culture. In group A, the NAAT(Gen-Probe) was unavailable, so we did not notify NAAT-positive cases. In group B, 102 cases were notified by positive NAAT(Gen-Probe) tests and finally 101 cases had positive MTB culture. By positive MTB culture, we notified 51 cases in Group A and 70 cases Group B respectively.

**Table 2:** Reasons for notification

Categories of notification	Group A	Group B
Positive AFB smear	431 (121)	13 (1)
Positive NAAT	0 (0)	102 (101)
Positive MTB culture	51 (51)	70 (70)
Total notified cases	482 (172)	185 (172)

The value in parentheses represent the numbers of confirmed PTB cases

**Fig. 2:**
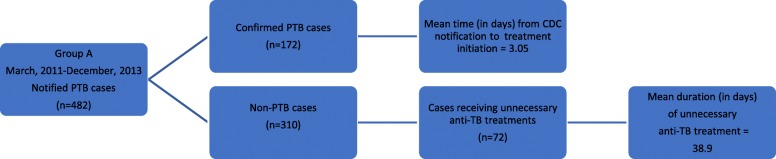
Group A was studied between March 2011 and December 2013, prior to the introduction of NAAT. * Notified PTB case: The case with suspected PTB reported to Taiwan CDC. * Confirmed PTB case: The case with positive MTB culture of the sputum. * Non-PTB case: The case with negative MTB culture of the sputum. * Unnecessary anti-TB treatment: Any TB medication administered in a notified case with negative result of the final culture, which is considered as prescription error. * Duration of unnecessary anti-TB treatment: The number of days of unnecessary TB medication administered in a notified case with negative result of the final MTB culture, which is considered as the duration of prescription error

From March 2011 to December 2013, notifications of 482 cases of suspected PTB were sent to Taiwan CDC (Group A) (Fig. 2). In this group, 172 cases of PTB were confirmed as positive for PTB based on positive MTB culture of the sputum. The results indicated that the rate of correct notification was 35.7%. Among the 172 cases of confirmed PTB, the mean time from CDC notification to treatment initiation was 3.05 days. The remaining 310 cases of Group A were confirmed as non-PTB based on negative MTB culture of the sputum. Error notification was defined as a case for which suspected PTB notification was sent to Taiwan CDC, but which showed a negative result on MTB culture. The rate of notification sent in error to CDC was 64.3% (Table 3). Of the 310 non-PTB cases, 72 patients (14.9%) received unnecessary anti-TB treatments. The mean duration of unnecessary TB medication for the 72 incorrectly diagnosed cases of PTB was 38.9 days.

**Table 3:** Main impact of NAAT(Gen-Probe)

Impact of NAAT	Group A	Group B	*P* value
Error notification rate	64.3%	7%	<0.001^a^
Unnecessary anti-TB treatments in notified cases	14.9%	6.5%	0.005^a^
Mean duration of unnecessary anti-TB treatments (d) (mean ± SD)	38.9 ± 38.3	37.0 ± 37.9	0.874^b^
Mean time from CDC notifications to treatment initiation (d) (mean ± SD)	3.05 ± 6.95	1.48 ± 1.99	0.004^b^

Abbreviations: *SD* standard deviation

^a^Using Chi-squared or Fisher’s exact tests (two-tailed)

^b^Using Wilcoxon rank-sum test

**Fig. 3:**
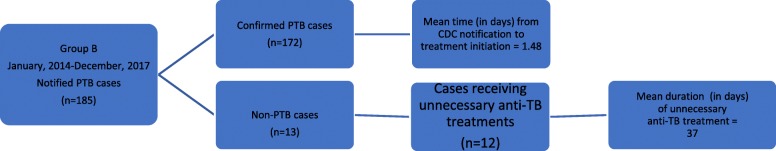
Group B was studied between January 2014 and December 2017, following the introduction of NAAT. * Notified PTB case: The case with suspected PTB reported to Taiwan CDC. * Confirmed PTB case: The case with positive MTB culture of the sputum. * Non-PTB case: The case with negative MTB culture of the sputum. * Unnecessary anti-TB treatment: Any TB medication administered in a notified case with negative result of the final culture, which is considered as prescription error. * Duration of unnecessary anti-TB treatment: The number of days of unnecessary TB medication administered in a notified case with negative result of the final MTB culture, which is considered as the duration of prescription error

During the period from January 2014 to December 2017, notifications of 185 suspected cases of PTB were sent by the hospital to Taiwan CDC (Group B; Fig. 3); in this group, 172 cases of PTB were confirmed as positive for PTB based on positive MTB culture of the sputum. The rate of correct notification sent to Taiwan CDC was 92.9%. In 172 cases of confirmed PTB, the mean time from notifying Taiwan CDC to initiating treatment was 1.48 days. Only 13 cases were confirmed as negative for PTB according to the final culture results. Hence, the rate of notification sent in error was 7.0% (Table 3). Of the 13 non-PTB cases, 12 patients (6.5%) received unnecessary anti-TB treatments. In 12 incorrectly diagnosed cases, the mean duration of unnecessary TB treatments was 37 days.

The impact of NAAT(Gen-Probe) is shown in Table 3. Following application of NAAT(Gen-Probe), the rate of error notification sent to Taiwanese CDC was decreased from 64.3 to 7.0% (*P* < 0.001), and the rate of unnecessary anti-TB treatments was decreased from 14.9 to 6.5% (*P* = 0.005); the mean duration of unnecessary anti-TB treatments was decreased from 38.9 days to 37.0 days, without significance (*P* = 0.874). In confirmed cases of PTB, the mean time from notifying Taiwan CDC to initiating treatment was decreased from 3.05 days to 1.48 days (*P* = 0.004).

The diagnostic performance of NAAT(Gen-Probe) is summarized in Table 4. The sensitivity, specificity, PPV, and NPV were 99.0, 92.3, 99.0, and 92.3%, respectively, and the area under curve for NAAT(Gen-Probe) was 0.96.

**Table 4:** Diagnostic performance of NAAT in group B

	NAAT
Sensitivity	99.0%
Specificity	92.3%
Positive predictive value	99.0%
Negative predictive value	92.3%

## Discussion

NAATs have revolutionized TB diagnosis. Several studies have indicated the impact of NAATs as a diagnostic tool that has been used in Taiwan for decades. The diagnostic performance of NAAT(Gen-Probe) in our study showed results similar to those of previous studies in Taiwan [3] and the US [13]. In Taiwan, Su et al. demonstrated the diagnostic performance of NAAT and compared with culture results, the sensitivity, specificity, PPV and NPV were 91.7, 98.6, 98.8 and 90.6%, respectively [3]. In the US, Laraque et al. reported that the sensitivity, specificity, PPV and NPV of NAAT, compared with culture results, were 91.7, 98.6, 98.8 and 90.6%, respectively [13]. Positive results on AFB smear are observed in both NTM colonization and Nocardia lung disease. Before the introduction of NAATs, a positive AFB smear resulted in unnecessary respiratory isolations [9, 10], treatments [9, 10], and contact investigations [10]. These precautions led to an increased waste of healthcare resources. The application of NAATs in positive-AFB smear specimens can reliably differentiate MTB from other organisms, thus avoiding unnecessary isolations. For example, integrating NAAT(Gen-Probe) into clinical decision making for patients with AFB-positive smears was associated with shorter mean time in airborne isolation (6.0 days vs 23.1 days) in the US [9]. Due to their high diagnostic performance and short turnaround time, NAATs have encouraged diagnosis of PTB at the community level and hospital, thereby accelerating early airborne isolation of inpatients and reducing nosocomial transmission in Taiwan [14].

PTB is a statutory communicable disease in Taiwan, and several measures have been undertaken to improve notification of the disease to public health officials. Physicians are required by regulation to notify Taiwan CDC of all suspected cases of PTB within 7 days or face a fine of 3000 to 15,000 USD for delayed notification. The TB registry in Taiwan has been in operation for decades. Following receipt of notification of suspected PTB, a public health official is required to visit all patients with suspected PTB within 7 days and complete a standard registration form. The TB registry records information of suspected or confirmed cases including the patient’s national identity number, birthdate, gender, result of bacteriological examination (AFB smear and MTB culture), site of disease (PTB or extrapulmonary TB), date of treatment initiation, treatment regimen, and patient outcome [15]. In the present study, NAAT(Gen-Probe) significantly decreased the rate of error notification, which dramatically decreased the workload of public health officials. To the best of our knowledge, this is the first study to report improvements in the accuracy of notification for cases of suspected PTB. Countries with high prevalence of PTB should employ NAATs to reduce the rate of error notification.

In a similar period why the number of cases has reduced to a large extent in Group B and How the culture positivity has increased in Group B of this study? Before the introduction of NAAT(Gen-Probe), physicians relied on positive AFB smear and positive MTB culture to notified PTB cases. In this era, physicians notified smear-positive cases to avoid disease transmission and penalty for delayed notification. But, most smear-positive cases (i.e. 310 cases / 431 cases = 71.9%) had negative MTB cultures. This resulted in reduced culture positivity (i.e. 172 cases/ 482 cases = 35.7%) and increased error notifications (i.e. 310 cases/ 482 cases = 64.3%). After the application of NAAT(Gen-Probe), smear-positive cases routinely received NAAT(Gen-Probe). Most smear-positive/NAAT-negative cases were not notified. Thus, NAAT(Gen-Probe) drastically reduced the numbers of notified cases (i.e. 482 cases – 185 cases = 297 cases). However, few smear-positive/NAAT-negative cases (i.e. 13 cases) were notified by some physician who were unfamiliar with the diagnostic performance of NAAT(Gen-Probe) at the beginning of NAAT(Gen-Probe) introduction. On the other hand, most cases (i.e. 172 cases / 185 cases = 93.0%) in group B were notified according to positive NAAT(Gen-Probe) test (i.e. 102 cases) and positive MTB culture (i.e. 70 cases). Owing to high positive predictive value (i.e. 101 cases / 102 cases = 99.0%) of NAAT(Gen-Probe), group B has increased culture positivity (i.e. 172 cases / 185 cases = 93.0%) and reduced error notifications (i.e. 13 cases / 185 cases = 7.0%). Taiwan has a high prevalence of TB and hepatitis caused by hepatitis B virus (HBV) and hepatitis C virus (HCV). In 2016, there were 10,328 cases of TB (43 cases per 100,000 population), and 547 TB-related deaths (2.3 cases per 100,000 population) [1]. Prior to initiating the national HBV vaccination program in 1984, approximately 15% of Taiwanese adults tested positive for HBV surface antigen [16]. An epidemiological study in 2007 has reported the seroprevalence of HCV in Taiwan of 4.4% [17]. Anti-TB medication is known to cause hepatotoxicity, and management of hepatitis in patients undergoing TB treatment who are co-infected with HBV or HCV is a challenge. A prospective study in Taiwan including 305 patients with TB [18] reported the prevalence rate of concomitant HBV and HCV infection was 11.7 and 6.7%, respectively; hepatitis during anti-tuberculous treatment (HATT), defined as an increase in serum transaminase level of > 3 times the upper limit of normal (ULN) with symptoms, or that of > 5 times the ULN without symptoms, developed in 68 patients (18.9%). Thus, PTB patients in Taiwan had high prevalence of HBV/HCV coinfection and were at high risk for drug-induced liver injury. Unnecessary TB medication should be avoided in countries with high prevalence of HBV/HCV. A retrospective study in Taiwan reported that 97 patients with suspected PTB based on initial AFB smear-positive sputum test were diagnosed as non-PTB cases according to the final results of mycobacterial culture and clinical judgment [19]; during the study period (2008–2011), NAATs were not available. In that study, of 97 patients with suspected PTB, as many as 25 patients (25.8%) received unnecessary TB medication, which highlighted the importance of NAATs to avoid wrong medication. In our study, NAAT(Gen-Probe) significantly decreased unnecessary administration of medication to patients with suspected TB. This finding represents a substantial improvement in public health, which may be relevant especially in countries where HBV and HCV are endemic. A study has investigated hypothetical reduction in unnecessary TB treatment by NAAT(Xpert) in the US; as a result, NAAT(Xpert) could avoid overtreatment of TB by 94% [20]. To the best of our knowledge, this was the first study to report the actual value of percentage reduction of unnecessary TB treatments by NAATs.

Several previous studies have indicated that NAATs shortened the duration of unnecessary TB medications. A retrospective study in the US showed that NAAT(Gen-Probe) reduced the mean duration of unnecessary anti-TB treatments by 9.5 days [9]. Another retrospective study in the US reported that outpatients for whom NAAT(Gen-Probe) was utilized, spent less time (average 1.5 months) taking unnecessary TB medications [10]. In the present study, NAAT(Gen-Probe) significantly decreased the percentage of patients receiving unnecessary TB treatments, but not the mean duration of unnecessary anti-TB treatments, which could be explained by the fact that the physicians were unfamiliar with NAAT(Gen-Probe) and relied on final culture results to terminate the unnecessary anti-TB treatments.

Delayed diagnosis and treatment of TB led to severe clinical disease and drug resistance [21, 22]. A time-period of > 60 days from the onset of TB symptoms to start of anti-TB treatment is associated with unfavorable treatment outcomes, including treatment failure and mortality in Ethiopia [23]. The BACTEC MGIT 960 System (BD, Franklin Lakes, NJ, USA) shortens the time required to culture mycobacteria from 6 weeks to 2 weeks [2]. However, substantial time is still required to confirm a diagnosis of airborne disease. Advances in NAAT(Xpert) have enabled the diagnosis of TB and simultaneous assessment of rifampicin resistance within 2 h of specimen collection [24]. Routine use of NAAT(Gen-Probe) has potential to reduce the overall turnaround time for laboratory diagnosis of TB by approximately 2 weeks [25]. Theoretically, NAATs can shorten the time taken to initiate treatments in patients with PTB. Several studies using different definitions of the starting time reported a reduced time to treatment initiation [11, 12]. Utilization of NAAT(Gen-Probe) has shown an impact on treatment decisions in California, USA [11]; the median time from specimen collection to initiation of TB treatments was shorter (3 days vs 14 days) with the use of NAAT (Gen-Probe). With regard to multidrug-resistant tuberculosis (MDRTB), a retrospective study in India has reported that the use of NAAT(Hain) reduced the median time from identification of patients suspected to have MDRTB to initiation of treatments from 157 days to 38 days [12]. In line with previous reports, our study indicated that NAAT(Gen-Probe) accelerated the mean time from CDC notification to treatment initiation. Hence, implementation of NAATs avoided treatment delay and improved the clinical outcomes. This was the first study to demonstrate that implementation of NAAT(Gen-Probe) hastened initiation of PTB treatment in Taiwan.

## Conclusion

NAAT(Gen-Probe) had a significant impact on the PTB notification system and treatments. NAATs provided accurate diagnosis of PTB by its high sensitivity and specificity. NAAT(Gen-Probe) improved the notification accuracy and enabled percentage reduction of unnecessary TB treatments in suspected cases of PTB. The use of NAATs shortened the time to treatment initiation. Physicians should consider using NAATs as a powerful tool during daily clinical practice.

### Limitations

Our study has limitations as follows. This retrospective study was limited to available data in the medical records. There were many potential factors that influenced the physicians’ decisions to initiate CDC notifications and PTB treatments. The impact of radiology findings or underlying disease was not elucidated. Treatment initiation may have been delayed in patients with liver cirrhosis. For positive AFB smear specimens, NAAT(Gen-Probe) was routinely performed on the same specimen; in contrast, for negative AFB smear specimens, NAAT(Gen-Probe) was not routinely performed. Because we only investigated the notified cases, the number of smear-positive/NAAT-negative cases which were not notified is beyond the scope of this study. Moreover, although culture is the gold standard, its sensitivity never reaches 100%; hence, the error notification is not 100% justified.

## Data Availability

The datasets analyzed during the current study are not publicly available because new search is being implemented but are available from the corresponding author on reasonable request. Then, the corresponding author will apply the request from the readers to the Institutional Review Board of Taipei Tzu Chi Hospital, Buddhist Tzu Chi Medical Foundation.

## Abbreviations

AFB: Acid fast bacilli
CDC: Centers for Disease Control and Prevention
HATT: Hepatitis during anti-tuberculous treatment
HBV: Hepatitis B virus
HCV: Hepatitis C virus
MDRTB: Multidrug-resistant tuberculosis
MGIT: Mycobacteria Growth Indicator Tube
MTB: *Mycobacterium tuberculosis*
NAAT: Nucleic acid amplification test
NAAT(Amplicor): Amplicor *Mycobacterium tuberculosis* test
NAAT(Gene-Probe): Enhanced Amplified *Mycobacterium tuberculosis* Direct Test
NAAT(Hain): Genotype MTBDR*plus* line probe assay
NAAT(Xpert): Xpert MTB/RIF Assay
NPV: Negative predictive value
NTM: Nontuberculous mycobacteria
PPV: Positive predictive value
PTB: Pulmonary tuberculosis
TB: Tuberculosis
ULN: Upper limit of normal
